# Pentaerythritol Tetranitrate In Vivo Treatment Improves Oxidative Stress and Vascular Dysfunction by Suppression of Endothelin-1 Signaling in Monocrotaline-Induced Pulmonary Hypertension

**DOI:** 10.1155/2017/4353462

**Published:** 2017-02-28

**Authors:** Sebastian Steven, Matthias Oelze, Moritz Brandt, Elisabeth Ullmann, Swenja Kröller-Schön, Tjebo Heeren, Lan P. Tran, Steffen Daub, Mobin Dib, Dirk Stalleicken, Philip Wenzel, Thomas Münzel, Andreas Daiber

**Affiliations:** ^1^Center for Cardiology, Department of Cardiology, Mainz, Germany; ^2^Center of Thrombosis and Hemostasis, University Medical Center Mainz, Mainz, Germany; ^3^Freelancer Consultant, 76199 Karlsruhe, Germany

## Abstract

*Objective*. Oxidative stress and endothelial dysfunction contribute to pulmonary arterial hypertension (PAH). The role of the nitrovasodilator pentaerythritol tetranitrate (PETN) on endothelial function and oxidative stress in PAH has not yet been defined.* Methods and Results*. PAH was induced by monocrotaline (MCT, i.v.) in Wistar rats. Low (30 mg/kg; MCT30), middle (40 mg/kg; MCT40), or high (60 mg/kg; MCT60) dose of MCT for 14, 28, and 42 d was used. MCT induced endothelial dysfunction, pulmonary vascular wall thickening, and fibrosis, as well as protein tyrosine nitration. Pulmonary arterial pressure and heart/body and lung/body weight ratio were increased in MCT40 rats (28 d) and reduced by oral PETN (10 mg/kg, 24 d) therapy. Oxidative stress in the vascular wall, in the heart, and in whole blood as well as vascular endothelin-1 signaling was increased in MCT40-treated rats and normalized by PETN therapy, likely by upregulation of heme oxygenase-1 (HO-1). PETN therapy improved endothelium-dependent relaxation in pulmonary arteries and inhibited endothelin-1-induced oxidative burst in whole blood and the expression of adhesion molecule (ICAM-1) in endothelial cells.* Conclusion*. MCT-induced PAH impairs endothelial function (aorta and pulmonary arteries) and increases oxidative stress whereas PETN markedly attenuates these adverse effects. Thus, PETN therapy improves pulmonary hypertension beyond its known cardiac preload reducing ability.

## 1. Introduction 

In humans, pulmonary arterial hypertension (PAH) is defined by a mean pulmonary arterial pressure of ≥25 mmHg at rest. It is a complex, multifactorial disease that involves endothelial dysfunction, oxidative stress, and remodeling of pulmonary vessels [1]. Concerning the pathophysiology of PAH the imbalance of the endothelial-derived vasoactive molecules nitric oxide (NO), prostacyclin (PGI_2_), superoxide (O_2_^•−^), and endothelin-1 (ET-1) plays a critical role [2–4]. Decreased bioavailability of NO, caused by increased formation of ROS, will result in an uncontrolled proliferation of smooth muscle cells (SMC), increased vascular tone, and therefore increased vascular resistance [5, 6]. Importantly, oxidative stress can stimulate both, the expressions of the ET-1 gene in endothelial cells and in SMC [7, 8]. Likewise, ET-1 signaling can trigger the production of reactive oxygen species (ROS) and inhibition of ET-1 receptor (A and B) by bosentan reduces oxidative stress in pulmonary hypertension [9].

In rodent models of PAH several enzymatic sources being responsible for increased ROS production have been identified such as the NADPH oxidase (Nox), xanthine oxidase (XO), an uncoupled endothelial NO synthase (eNOS), and mitochondria [10–14]. In patients suffering from PAH, increased oxidative stress and XO activity have been demonstrated [15]. 3-nitrotyrosine (3-NT) levels, a foot print for in vivo formation of the NO and superoxide (O_2_^•−^) reaction product peroxynitrite (ONOO^−^), have been shown to be increased in lung biopsy samples of patients suffering from PAH [16]. These data clearly indicate that lungs of PAH patients are exposed to chronic oxidative stress likely leading to reduced NO bioavailability [15, 16].

Organic nitrates, given acutely, are potent NO dependent vasodilators used for the treatment of chronic stable angina and unstable angina, myocardial infarction, congestive heart failure, and arterial hypertension [17]. Although organic nitrates are theoretically beneficial in PAH, because of their properties as a vasodilator, they have been used with limited clinical results because of the lack of pulmonary selectivity [18]. Therefore, inhaled NO and nitroglycerin (NTG) were tested for the treatment of PAH [19–21]. In experimental MCT-induced PAH, the organic nitrate and K^+^-channel opener, nicorandil, showed beneficial effects: right ventricular systolic pressure (RVSP) was decreased, accompanied by higher expression of eNOS and less endothelial damage [22].

Pentaerythritol tetranitrate (PETN; IUPAC 2,2-Bis[(nitrooxy)methyl]propane-1,3-diyl dinitrate, Figure 3(a)) is an organic nitrate with fewer side effects on the vasculature than other nitrates like NTG, isosorbide-5-mononitrate (ISMN), or isosorbide dinitrate (ISDN) [17, 23, 24]. In humans PETN causes side effects like headache, hypotension, or tachycardia. However, the therapeutic range is broad since LD_50_/24 h in rats is >900 mg/kg [25, 26]. Due to its limited solubility in water (43 mg/L at 25°C) oral treatment is preferably performed by diet [27]. In contrast to ISMN or NTG, PETN improved vascular function in animal models of diabetes and hypertension [27, 28]. The beneficial effects and favorable side effects profile of PETN have been attributed to the induction of the antioxidant enzymes heme oxygenase-1 (HO-1), ecSOD, and ferritin [28–32].

Since PAH is improved by HO-1 induction [33, 34], PETN is a potential candidate drug for treatment of this severe disease, currently investigated within the CAESAR clinical trial (“ClinicAl Efficacy Study of Pentalong® for PulmonAry Hypertension in HeaRt Failure”; EudraCT Number: 2009-015059-26). We therefore focused on the influence of the organic nitrate PETN on pulmonary arterial pressure, vascular function, and oxidative stress in MCT-induced PAH.

## 2. Methods

For isometric tension studies, NTG was obtained from a Nitrolingual infusion solution (1 mg/mL) from G. Pohl-Boskamp (Hohenlockstedt, Germany). The Bradford reagent was obtained from BioRad (Munich, Germany). PETN (20%)/lactose (80%) mixture was a kind gift of Actavis Deutschland GmbH (now PUREN Pharma GmbH & Co. KG, Munich, Germany). There is no measurable degradation of PETN when stored dry and solid but in this state PETN is pressure-sensitive and highly explosive. For this reason PETN is stored and shipped as a lactose mixture (usually >80 w/w% lactose). The solubility of PETN in DMSO is excellent: stock solutions of >100 mM are easy to prepare and can be stored for months at −20°C without serious loss of PETN. In aqueous solutions PETN is slowly hydrolyzed over days. All other chemicals were obtained from Fluka, Sigma-Aldrich, or Merck.

### 2.1. Animals and In Vivo Treatment

90 male Wistar rats (8 weeks, 250 g) were used for the experiments. All animals were treated in accordance with the Guide for the Care and Use of Laboratory Animals as adopted by the US National Institutes of Health. Approval was granted by the Ethics Committee of the University Hospital Mainz and the Landesuntersuchungsamt (#23 177-07/G 10-1-039). Monocrotaline (MCT) injection was used as a model for pulmonary arterial hypertension. For characterization of the model, isometric tension studies were performed 2, 4, and 6 weeks after single i.v. injection of 30, 40, and 60 mg/kg body weight MCT into the* vena dorsalis penis*. In regard to effectiveness of PAH induction and mortality of the animals, the 40 mg/kg MCT dose for 4 weeks was chosen for experiments with the organic nitrate and PETN (10 mg/kg/day, p.o.) therapy by diet (Ssniff Spezialdiäten, Soest, Germany) was started 3 days after MCT injection. The control and MCT group received standard diet without PETN. After 3.5 weeks of PETN treatment, rats were killed by exsanguination in isoflurane anaesthesia, and the blood, aorta, pulmonary artery, and heart were collected.

### 2.2. Isometric Tension Recordings

Perivascular fat was removed from every aorta and pulmonary arteries. Concentration-relaxation curves in response to increasing concentrations acetylcholine (ACh) were performed as described [35].

### 2.3. Histological Staining of Lung Tissue

Trichrome staining (according to Oelze et al.) was performed with paraffin-embedded samples of lung tissue upon deparaffination as described [36]. Afterwards the nuclei were prestained with haematoxylin (according to Meloan and Puchtler) [37]. Then samples were stained for 5 minutes with Mallory red containing 100% acetic acid, fuchsine acid, and Orange G (Merck, Darmstadt, Germany), then for 15 minutes in 1% molybdatophosphoric acid hydrate (VWR, Darmstadt, Germany), and then for 5 minutes in acid light green. Finally tissue samples were dehydrated in glacial acetic acid and 100% ethanol and coverslipped in Entellan®.

### 2.4. Determination of Heart to Body (h/b) and Lung to Body (l/b) Weight Ratio

Heart to body and lung to body weight ratio were determined by weighing animals prior to sacrifice. After organ removal weight of heart and lung was measured. Every individual body weight was set into relation to heart and lung weight of the animal.

### 2.5. Small Animal Echocardiography

Anaesthesia of rats was induced in a chamber (2–4% isoflurane mixed with 0.2 L/min 100% O_2_) and maintained with a face mask (1-2% isoflurane with 0.2 L/min 100% O_2_). Animals were kept on a heated table mounted on a rail system (Visual Sonics, Toronto, Canada). Ultrasound was performed with the Vevo 770 System and a 25 MHz transducer (VisualSonics). Heart rate was monitored; body temperature was monitored using a rectal probe and maintained at 37°C. Two-dimensional images of the pulmonary valve were obtained from the parasternal short-axis view at the level of the aortic valve and pulsed-wave Doppler recordings of the blood flow at the tips of the cusps of the pulmonary valve were obtained with the beam oriented parallel to the flow. The sweep speed for the Doppler flow recordings was 400–800 mm/s. Pulmonary arterial acceleration time (PAT) was measured and systolic pulmonary arterial pressure (sPAP) was calculated as described [38].

### 2.6. Dot Blot and Western Blot Analysis

3-nitrotyrosine positive proteins were assessed by dot blot analysis [28]. For detection of 3-NT, a primary mouse monoclonal nitrotyrosine antibody (Millipore, Billerica, USA) was used at a dilution of 1 : 1000. Detection and quantification were performed by ECL with peroxidase conjugated secondary antibodies against mouse (1 : 10000, Vector Lab., Burlingame, CA). Densitometric quantification of antibody-specific dots was performed with a ChemiLux Imager (CsX-1400M, Intas, Göttingen, Germany) and Gel-Pro Analyzer software (Media Cybernetics, Bethesda, MD).

Isolated aortic and pulmonary tissue from rat was frozen in liquid nitrogen and homogenized in buffer (Tris-HCl 20 mM, saccharose 250 mM, ethylene glycol-bis(*β*-aminoethyl ether)-N,N,N′,N′-tetraacetic acid (EGTA) 3 mM, ethylene diamine tetraacetic acid (EDTA) 20 mM, protease inhibitor cocktail (Roche complete, 1 tablet in 100 mL), and Triton-X-100 1 v/v%). Proteins were separated by SDS-Page and blotted onto nitrocellulose membranes [23]. After blocking, immunoblotting was performed with the following antibodies: monoclonal mouse *β*-actin (42 kDa) as a control for loading and transfer and polyclonal mouse NADPH oxidase 2 (Nox2, 1 : 500, BD Bioscience, USA). Detection and quantification were performed by ECL with peroxidase conjugated anti-mouse (1 : 10,000, Vector Lab., Burlingame, CA). Densitometric quantification as described above.

### 2.7. Detection of Oxidative Stress in Cardiac Membrane Fractions, Pulmonary Arteries, Aortic Vessels, and Serum and Blood Samples

Reactive oxygen species formation was measured by oxidative burst of leukocytes in whole blood (stimulated with the phorbol ester PDBu, 10 *µ*M) or NADPH oxidase activity in the heart by ECL (100 *µ*M L-012 and 5 *µ*M lucigenin plus 200 *µ*M NADPH, respectively) [39]. For ROS formation in pulmonary vessels, isolated pulmonary artery rings were OCT-embedded (Tissue Tek, USA) and upon staining with dihydroethidium (DHE, 1 *µ*M) oxidative fluorescence microtopography was determined as reported [40]. Xanthine oxidase activity was measured in serum, which was diluted 1 : 1 with cytochrome c (100 *μ*M) in PBS containing either hypoxanthine (1 mM) or allopurinol (1 mM) [41]. The superoxide-driven reduction of cytochrome c was measured by the absorption of ferrous cytochrome c at 550 nm as the difference between hypoxanthine and allopurinol containing buffer. Superoxide formation rates were calculated using *ε*_550_ = 19,500 mM^−1^ cm^−1^ for reduced cytochrome c. Total serum antioxidant capacity was measured by the reduction of the stabilized 2,2-diphenyl-1-picrylhydrazyl radical (DPPH^•^) (50 *µ*M) by serum antioxidants tracing the absorption at 517 nm [27].

### 2.8. Reverse Transcription Real-Time PCR (qRT-PCR)

mRNA expression was analyzed with quantitative real-time RT-PCR as previously described [42]. Briefly, total RNA from rat lung was isolated (RNeasy Fibrous Tissue Mini Kit; Qiagen, Hilden, Germany), and 50 ng of total RNA was used for real-time RT-PCR analysis with the QuantiTectTM Probe RT-PCR kit (Qiagen). TaqMan® Gene Expression assays for heme oxygenase (HO-1), vascular adhesion molecule-1 (VCAM-1), endothelin-1 (ET-1), endothelin-1 a receptor, endothelin-1 b receptor, endothelin converting enzyme-1 (ECE-1), intercellular adhesion molecule-1 (ICAM-1), and TATA box binding protein (TBP) were purchased as probe-and-primer sets (Applied Biosystems, Foster City, CA). The comparative Ct method was used for relative mRNA quantification. Gene expression was normalized to the endogenous control (TBP mRNA), and the amount of target gene mRNA expression in each sample was expressed relative to that of control.

### 2.9. Chemiluminescence-Based Detection of Oxidative Burst of Leukocytes in Whole Blood

Human samples were obtained and handled in accordance with the Declaration of Helsinki and our institutional ethical guidelines. Whole blood was obtained from at least four different healthy volunteers by vein puncture. Oxidative burst mainly reflects NADPH oxidase (Nox) and myeloperoxidase activity and was therefore used as a read-out for the degree of activation of white blood cells in whole blood. Briefly, blood was incubated with PETN, stimulated with the ET-1 analog BQ-3020 (0.05 *µ*M, 0.5 *µ*M, and 5 *µ*M), and ROS formation was assessed in PBS containing Ca^2+^/Mg^2+^ (1 mM) by L-012 (100 *µ*M) enhanced chemiluminescence (ECL).

### 2.10. ICAM-1 Expression in Cultured Endothelial Cells

The human endothelial cell line EA.hy 926 was a gift from C.-J. S. Edgell (University of North Carolina at Chapel Hill, USA). EA.hy 926 cells were grown at 10% CO_2_ in Dulbecco's modified Eagle's medium (DMEM, Sigma) with 10% fetal calf serum, 2 mM l-glutamine, 1 mM sodium pyruvate, 100 IU/mL penicillin, and 100 *μ*g/mL streptomycin. Semiconfluent cells (6-well plates) were used for further experiments. Cultured endothelial cells (Ea.hy) were incubated with 5 *µ*M BQ3020 (endothelin-1 analog), 100 *µ*M PETN, or solvent (DMSO). After 24 hours cells were lyzed in GIT-buffer (guanidinium isocyanate, sodium-citrate, and N-lauroylsarcosine) and mRNA was isolated by phenol extraction.

### 2.11. Statistical Analysis

Results are expressed as mean ± SEM. Two-way ANOVA (with Bonferroni's correction for comparison of multiple means) was used for comparisons of vasodilator potency and efficacy and kinetic traces of whole blood oxidative burst. *T*-test was used for comparison of MCT-induced changes in endothelial function (expressed as changes in efficacy (maximal relaxation)) between two groups. One-way ANOVA (with Bonferroni's or Dunn's correction for comparison of multiple means) was used for comparisons of heart/body and lung/body weight, echocardiography, blood, cardiac, aortic, and whole blood ROS formation, and protein and mRNA expression. *p* values < 0.05 were considered as statistically significant.

## 3. Results

### 3.1. MCT Induces PAH, Pulmonary Fibrosis, and Nitro-Oxidative Stress

**Figure 1:**
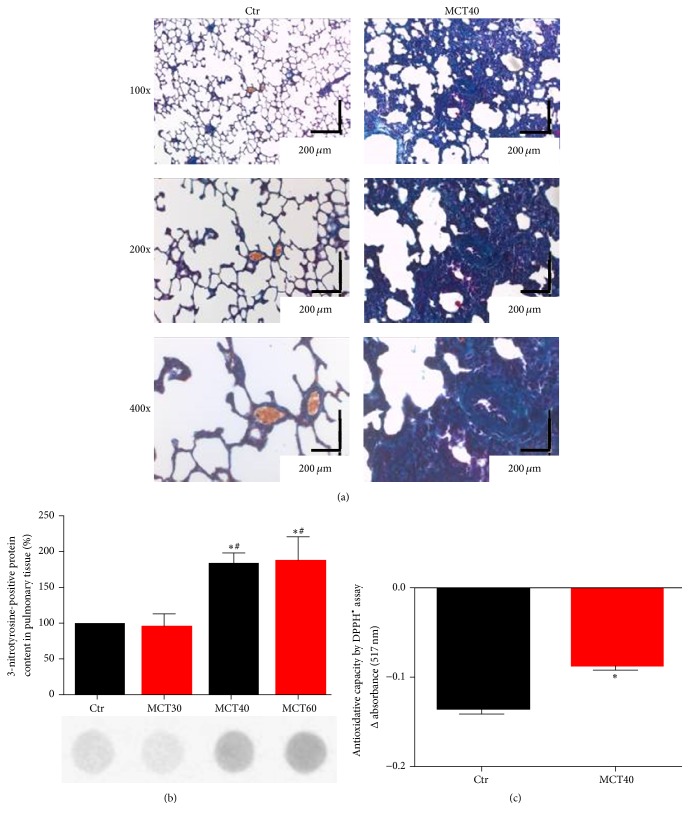
Characterization of morphological changes and protein nitration in lung tissue and serum antioxidant capacity of monocrotaline-treated rats. (a) Trichrome staining of paraffinated lung tissue (magnification of 100x, 200x, and 400x). (b) Levels of 3-NT positive proteins in lung tissue were assessed by dot blot analysis and specific antibodies. Representative blots are shown below the densitometric quantification. (c) Antioxidant capacity was determined by DPPH^•^ assay (ΔE 517 nm). The data are mean ± SEM from 3–8 (a, b) and 3–6 (c) animals/group. ^*∗*^*p* < 0.05 versus control and ^#^*p* < 0.05 versus MCT30.

Four weeks following MCT40 treatment, PAP of rats was significantly increased up to 53.96 ± 5.28 mmHg versus 26.63 ± 2.26 mmHg in controls (*p* < 0.05). PAH caused vascular wall thickening in small and medium sized vessels as well as fibrosis (Figure 1(a)). MCT40 and MCT60 treatment significantly increased staining of 3-NT positive proteins in the lungs, while MCT30 showed no difference to control (Figure 1(b)). The total antioxidant capacity in serum was significantly decreased by MCT40 (Figure 1(c)).

### 3.2. Effects of MCT-Induced PAH on Vascular Function of Aorta and Pulmonary Arteries

**Figure 2:**
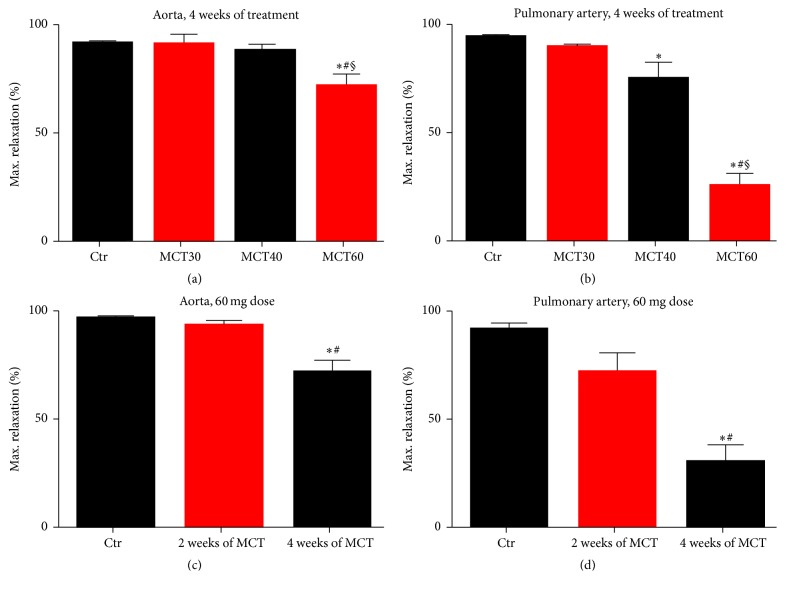
Characterization of vascular function in the model of monocrotaline-induced pulmonary hypertension. Endothelium-dependent (ACh) maximum relaxation was determined by isometric tension studies in aortic ring segments (a, c) and pulmonary ring segments (b, d) from rats treated with different doses of MCT and different time protocols for the induction of pulmonary hypertension. A total number of 4–22 (a, b) and 6–14 (c) and 6 (d) aortic and pulmonary artery ring segments from at least 3 male rats were used. ^*∗*^*p* < 0.05 versus control (a–d); ^#^*p* < 0.05 versus MCT30 (a, b) or 2 weeks (c, d); ^§^*p* < 0.05 versus MCT40 (a, b).

MCT treatment impaired endothelium-dependent relaxation of the aorta to ACh dose-dependently, with a significant difference in the MCT60 group compared to controls (Figure 2(a)). Also endothelial function of pulmonary arteries was significantly impaired in response to middle and high dose of MCT (Figure 2(b)). To further characterize the MCT-induced pulmonary hypertension model, vascular function in response to the highest MCT60 dose was tested in a time-dependent fashion (2 and 4 weeks, longer treatment (for 6 weeks) resulted in significant (>50%) mortality of the animals). Endothelium-dependent vasodilation in aorta (Figure 2(c)) and pulmonary arteries (Figure 2(d)) was examined 2 and 4 weeks after MCT administration. Vascular function was impaired significantly in response to 4 weeks of treatment for aorta and all treatment durations for pulmonary arteries. The dose of MCT40 (40 mg/kg for 4 weeks) was used for all other experiments.

### 3.3. Effects of PETN on Pulmonary Arterial Pressure and Cardiac and Lung/Pulmonary Artery Hypertrophy in PAH

**Figure 3:**
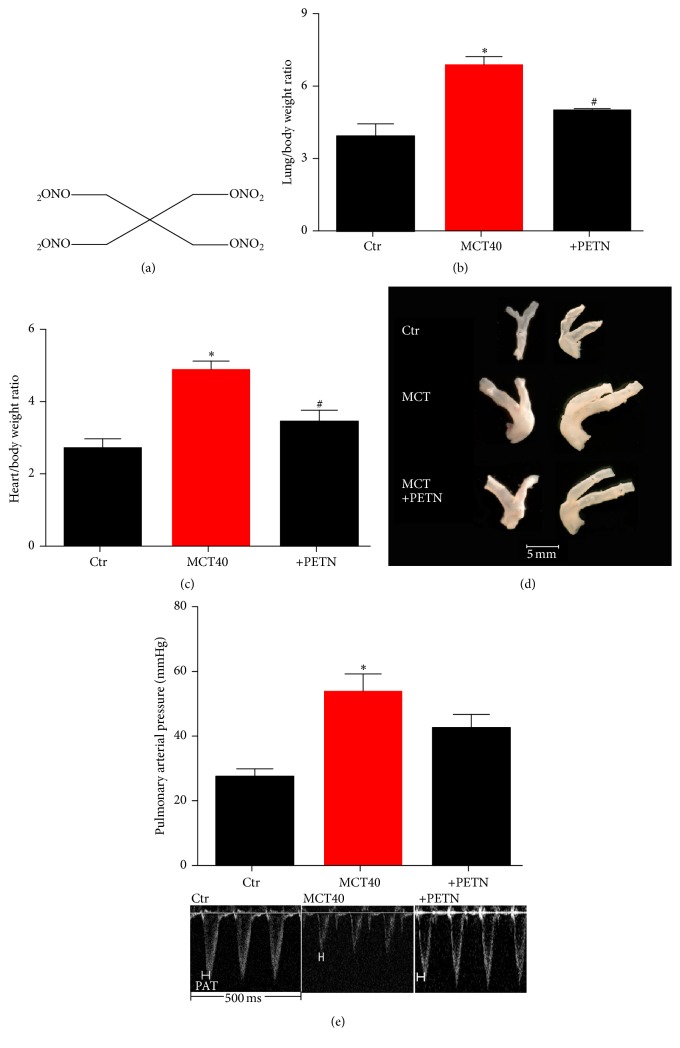
Effects of PETN therapy on heart/body and lung/body weight ratio, pulmonary artery dilation, and pulmonary arterial pressure in monocrotaline-treated rats (MCT40, 4 weeks). (a) Chemical structure of PETN. (b) Lung/body and (c) heart/body weight ratio were determined. (d) Pulmonary artery dilation was qualitatively envisaged by photographic images. (e) Echocardiography was used to measure pulmonary arterial pressure. The data are mean ± SEM of 4 (b, c) and 3-4 (d, e) animals per group ^*∗*^*p* < 0.05 versus control and ^#^*p* < 0.05 versus MCT.

Heart to body (h/b) and lung to body (l/b) weight ratio of MCT40 treated rats were increased as a sign for cardiac hypertrophy due to high cardiac afterload and pulmonary hypertension (Figures 3(b) and 3(c)). Additionally, pulmonary arteries were dilated (Figure 3(d)). The organic nitrate PETN significantly improved these morphological changes. PETN treatment prevented a further significant increase in PAP in the MCT40 group (Figure 3(e)).

### 3.4. Effects of PETN on Vascular Function of Pulmonary Arteries and Nitro-Oxidative Stress in PAH

**Figure 4:**
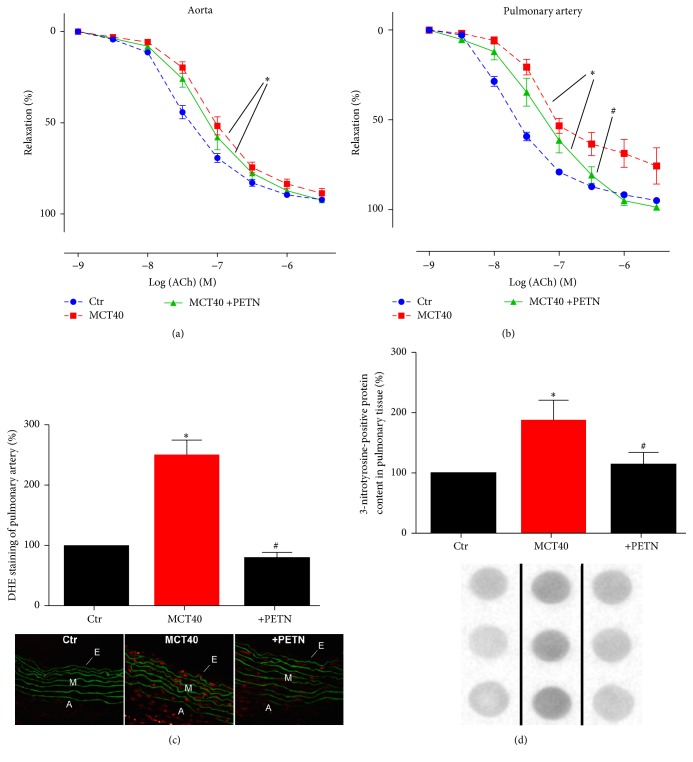
Effects of PETN therapy on endothelial function, oxidative stress, and protein tyrosine nitration of aorta and pulmonary artery in monocrotaline-treated rats (MCT40 4 weeks). (a, b) Endothelium-dependent (ACh) relaxation was determined by isometric tension studies in rat aortic ring segments and pulmonary ring segments. (c) DHE (1 *µ*M) oxidative fluorescence microtopography was used to assess vascular oxidative stress. (d) Levels of 3-NT positive proteins in lung tissue were assessed by dot blot analysis and specific antibodies. Representative blots are shown below the densitometric quantification. A total number of 6–22 aortic (a) and 6–17 pulmonary tissue (b–d) ring segments from male rats were used. ^*∗*^*p* < 0.05 versus control and ^#^*p* < 0.05 versus MCT40.

PETN did not significantly improve PAH induced endothelial dysfunction of the aorta (Figure 4(a)) while ameliorating endothelial function of pulmonary vessels in the PAH group (MCT40) (Figure 4(b)). DHE fluorescence increased throughout the wall (endothelium, media, and adventitia) of pulmonary arteries in the setting of PAH and was normalized by PETN (Figure 4(c)). The content of 3-NT positive proteins in lung tissue, determined by dot blot analysis, was significantly increased in the PAH group and reduced by PETN (Figure 4(d)).

### 3.5. Effects of PETN Therapy on Systemic Oxidative Stress in PAH

**Figure 5:**
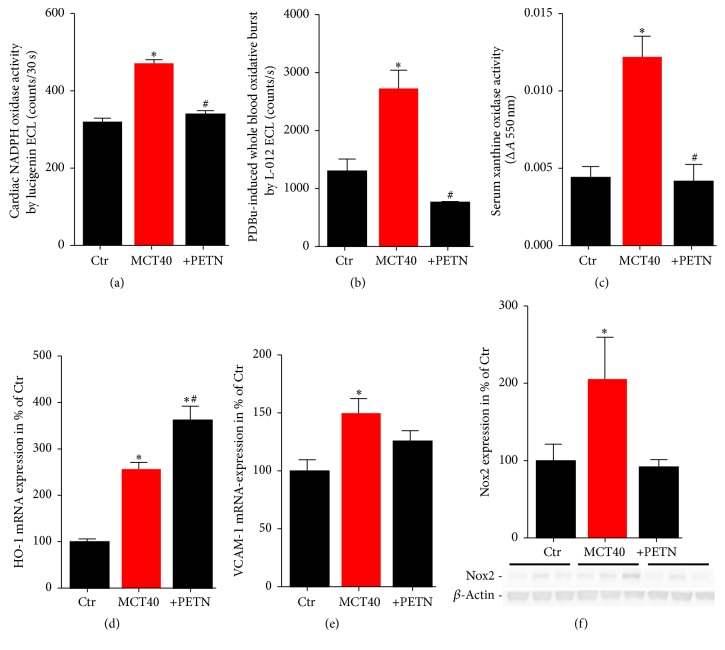
Effects of PETN therapy on prooxidative protein activity (Nox, XO), oxidative burst in whole blood, and antioxidant HO-1 mRNA expression in pulmonary hypertension (MCT40, 4 weeks). (a) Cardiac NADPH oxidase (Nox) activity was measured by the chemiluminescence probe lucigenin (5 *µ*M) in the presence of NADPH (200 *µ*M). (b) Leukocyte-derived oxidative burst in whole blood was examined by the chemiluminescence probe L-012 (100 *µ*M) upon stimulation with PDBu (10 *µ*M). (c) Xanthine oxidase (XO) activity was assessed by a photometric assay using cytochrome c (change in absorbance: ΔA 550 nm). qRT-PCR was used to determine mRNA expression levels of the antioxidant enzyme (d) heme oxygenase-1 (HO-1) and (e) vascular adhesion molecule-1 (VCAM-1) in lung tissue. (f) NADPH oxidase 2 (Nox2) protein expression was determined by Western blot analysis. The data are mean ± SEM from 3–6 animals/group. ^*∗*^*p* < 0.05 versus control; ^#^*p* < 0.05 versus MCT40.

Cardiac NADPH oxidase (Nox) activity, which was markedly increased in PAH, was completely normalized by PETN treatment (Figure 5(a)), which was supported by Nox2 protein expression showing a similar pattern (Figure 5(f)). The oxidative burst measured in whole blood (as a read-out for phagocytic NADPH oxidase activity) was increased in MCT40 rats and normalized in the PETN group (Figure 5(b)). Furthermore, activity of xanthine oxidase (XO) was increased by MCT treatment and completely normalized by PETN therapy (Figure 5(c)). mRNA levels of the antioxidant enzyme HO-1 in lung tissue were significantly increased in pulmonary hypertension and the administration of PETN led to an additional increase in HO-1 expression (Figure 5(d)). It might be speculated that prevention of the nitro-oxidative stress by PETN therapy also ameliorated the inflammatory phenotype in the MCT40 animals as demonstrated by assessment of VCAM-1 mRNA expression (Figure 5(e)).

### 3.6. Effects of PETN Therapy on Endothelin-1 Signaling in PAH

**Figure 6:**
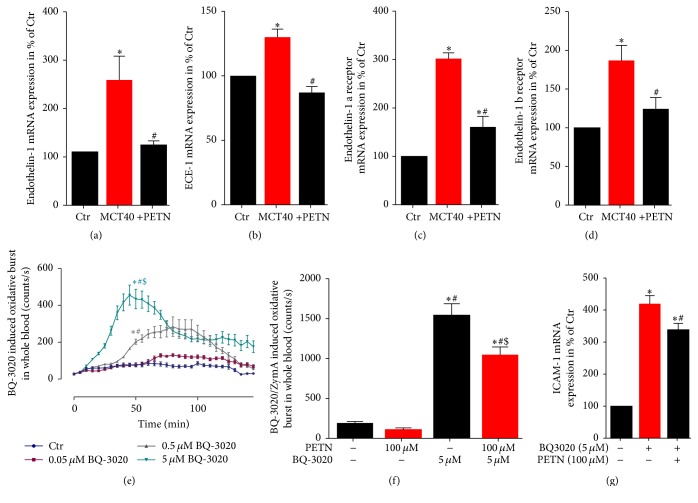
Effects of PETN therapy on endothelin-1 signaling (ET-1, ECE-1, and ET-1a/b receptor) and oxidative burst in whole blood. qRT-PCR was used to determine mRNA expression levels of (a) endothelin-1, (b) endothelin-1 converting enzyme-1, (c) endothelin-1 a receptor, and (d) endothelin-1 b receptor in lung tissue. (e) Leukocyte-derived oxidative burst in whole blood was examined by the chemiluminescence probe L-012 (100 *µ*M) upon stimulation with the endothelin-1 agonist BQ-3020 (0.05–5 *µ*M). (f) Effect of PETN (100 *µ*M) on BQ-3020 (5 *µ*M)/zymosan A (0.5 *µ*g/mL) stimulated whole blood oxidative burst. (g) qRT-PCR was used to determine mRNA expression levels of ICAM-1 in EA.hy cells upon stimulation with BQ-3020 and PETN. The data are mean ± SEM from 6 animals/group (a–d) and 16 (e-f) or 3–7 (g) independent experiments. ^*∗*^*p* < 0.05 versus control; ^#^*p* < 0.05 versus MCT40.

In MCT-treated groups the pulmonary mRNA expression levels of ET-1, endothelin converting enzyme (ECE-1), ET_A_ (ET-1a), and ET_B_ (ET-1b) receptor were significantly increased compared to healthy control rats. Treatment of pulmonary hypertension with PETN led to a normalization of ET-1, ECE-1, ET-1a, and ET-1b mRNA expression levels (Figures 6(a)–6(d)). Incubation of human whole blood with the ET-1 analog BQ-3020 induced a concentration-dependent increase of oxidative burst (Figure 6(e)), whereas PETN incubation significantly reduced the oxidative burst in BQ-3020/zymosan A-stimulated blood (Figure 6(f)). Furthermore, longer incubations (24 hours) with the ET-1 analog BQ-3020 induced adhesion molecule (ICAM-1) mRNA expression in cultured endothelial cells (EA.hy), which was ameliorated by PETN coincubation (Figure 6(g)).

## 4. Discussion

The results of the present study demonstrate that the organic nitrate PETN reduces oxidative stress and improves endothelial function of pulmonary arteries in monocrotaline-induced PAH. We identified modulation of heme oxygenase-1 expression and endothelin-1 signaling to be responsible for the beneficial effects of PETN in PAH. Recent treatment guidelines recommend treatment of PAH with macitentan (endothelin-1 receptor blocker), sildenafil (PDE5-inhibitor), iloprost (prostacyclin analog), or newly introduced riociguat (sGC stimulator) [43]. All compounds improve PAH mainly via dilation of the pulmonary vessels and therefore reduction of pulmonary vascular resistance (PVR). A vasodilating drug with additional anti-inflammatory and antioxidant properties could be a useful tool to improve therapy of PAH. Additionally, PETN was shown to reduce ET-1 plasma levels in human, which might be another approach for protective effects in PAH [44].

MCT is a toxic alkaloid from* Crotalaria spectabilis *and was used for induction of PAH. In the MCT PAH-model vascular wall thickening and pulmonary inflammation involving neutrophil infiltration lead to high afterload of the right ventricle, which consequently results in cardiac hypertrophy [45]. Antioxidant capacity in serum of MCT-treated animals was reduced and 3-nitrotyrosine-positive protein content in pulmonary vessels was increased dependent on the MCT dose used. Accordingly, endothelium-dependent relaxation not only in pulmonary arteries but also in aorta was impaired in a MCT-dose dependent fashion. Investigations on the effect of the natural phenol and antioxidant resveratrol on MCT-induced pulmonary hypertension revealed the important role of oxidative stress in PAH [10]. In pulmonary hypertensive rats, resveratrol attenuated right ventricular blood pressure, pulmonary artery remodeling (SMC proliferation), and pulmonary inflammation (reduced leukocyte infiltration). The latter study as well as other preclinical and clinical investigations on pulmonary hypertension underlines the role of oxidative stress in the pathogenesis of PAH [15, 16, 46–48].

As we demonstrated before, the organic nitrate PETN is different to other nitrates like nitroglycerin (NTG) or isosorbide dinitrate (ISDN) [17, 49, 50]. Acute treatment with organic nitrates such as NTG has potent vasodilator and anti-ischemic effects in patients with acute coronary syndromes, congestive heart failure, and arterial hypertension. However, long-term treatment is associated with nitrate tolerance and endothelial dysfunction, which reduces the therapeutic efficacy of these drugs. PETN seems to be different and human studies in healthy volunteers and patients with coronary artery disease showed preserved vasodilatatory potency and no induction of oxidative stress or endothelial dysfunction [51–53]. Also animal studies revealed a prevention of endothelial dysfunction as well as progression of vascular lesions in established atherosclerosis by PETN [54], which might be mediated by upregulation of the antioxidant defense protein HO-1 [55]. Recently we demonstrated that HO-1 is a regulator of vascular function in arterial hypertension via modulation of inflammatory monocytes [56] and we showed HO-1 expression to be induced by PETN in a rat model for type 1 diabetes and arterial hypertension [27, 28]. HO-1 induction seems to be a potent mechanism to reduce oxidative stress and tissue inflammation, not only in arterial, but also in pulmonary hypertension. Shimzu et al. showed attenuation of pulmonary hypertension and reduced pulmonary inflammation by HO-1 induction using hemin [33]. HO-1 catalyzes the degradation of heme into biliverdin (which is converted to the antioxidant bilirubin), the gaseous transmitter carbon monoxide (CO), and free iron, the latter leading to upregulation of ferritin and reduced free iron levels in the long run. Therefore, HO-1 induction is considered as an essential physiological stress response pathway conferring antiatherosclerotic and beneficial effects on endothelial function [27, 28, 57]. Here we found an additive increase in HO-1 mRNA expression by PETN in lung tissue of PAH rats providing an attractive explanation for the beneficial effects of PETN therapy on vascular and pulmonary oxidative stress parameters and subsequent improvement of endothelial function in pulmonary arteries, PAP, and morphological changes (heart/body, lung/body ratio) observed in MCT-treated animals.

Chen et al. demonstrated increased iNOS expression in MCT-induced PAH and ROS levels are known to be elevated in PAH [46, 58]. This can be explained by monocyte/macrophage extravasation in pulmonary tissue and increased iNOS expression by these cells. Since Nox-derived ROS are known to participate in the pathogenesis of pulmonary hypertension, increased 3-NT positive proteins indicate vascular inflammation and Nox activation. On the other hand, elevated ROS levels react with eNOS-derived NO to ONOO^−^, which reduces NO bioavailability and thereby contributes to endothelial dysfunction. In turn, reduced NO bioavailability explains not only the observed endothelial dysfunction, but also smooth muscle cell (SMC) proliferation and fibrosis in small and medium sized pulmonary vessels as previously reported [6]. We assessed nitro-oxidative stress levels in lung tissue by 3-NT positive protein content reflecting peroxynitrite (ONOO^−^) formation and DHE fluorescence microtopography in pulmonary arteries. Both nitro-oxidative stress parameters were clearly increased in MCT-treated animals and normalized by PETN therapy.

Endothelin-1 is known to play an essential role in the pathogenesis of PAH [3] and blockade of the ET-1 receptor with macitentan or bosentan is an established treatment option for PAH underlining the relevance of ET-1 signaling in PAH. Furthermore, it has been shown that oxidative stress leads to increased endothelial synthesis of endothelin-1 [8]. In this study, PETN therapy downregulated ET-1, ECE-1, and ET-1a/b receptor mRNA expression in MCT-induced pulmonary hypertension. Furthermore, PETN suppressed ET-1 (BQ-3020) dependent oxidative burst in whole blood and normalized ICAM-1 mRNA expression in cultured endothelial cells (Ea.hy). It might be speculated that the beneficial effects of PETN on ET-1 signaling are mediated by suppression of oxidative stress, a potent trigger of ET-1 signaling.

## 5. Conclusions

PETN improves to a minor extent vascular remodeling and endothelial function and more potently suppresses oxidative stress as well as pathological changes in heart/body and lung/body weight ratio in an experimental model of PAH by induction of HO-1 but also by interference with ET-1 signaling. The MCT model clearly demonstrates some limitations as a model of clinical PAH, especially since morphological changes develop quite fast and are not easily prevented by therapeutical interventions. Most importantly, clinical data are needed to proof our experimental findings. The ongoing CAESAR clinical trial (“ClinicAl Efficacy Study of Pentalong for PulmonAry Hypertension in HeaRt Failure”) will answer the question, whether PETN represents a new option for the treatment of patients with PAH.

## Abbreviations

3-NT: 3-nitrotyrosine
ACh: Acetylcholine
DHE: Dihydroethidium
ecSOD: Extracellular superoxide dismutase
eNOS: Endothelial nitric oxide-synthase
ET-1: Endothelin-1
ET-1a: Endothelin-1 receptor type a
ET-1b: Endothelin-1 receptor type b
h/b ratio: Heart to body weight ratio
HO-1: Heme oxygenase-1
i.v.: Intravenously
ICAM-1: Intercellular adhesion molecule 1
ISDN: Isosorbide dinitrate
MCT: Monocrotaline
MCT30: 30 mg monocrotaline
MCT40: 40 mg monocrotaline
MCT60: 60 mg monocrotaline
NO: Nitric oxide
Nox: NADPH oxidase
NTG: Nitroglycerin
O_2_^•−^: Superoxide
PAH: Pulmonary arterial hypertension
PAP: Pulmonary arterial pressure
PETN: Pentaerythritol tetranitrate
PGI_2_: Prostacyclin
PVR: Pulmonary vascular resistance
ROS: Reactive oxygen species
RVSP: Right ventricular pressure
s.c.: Subcutaneously
SMC: Smooth muscle cell
VCAM-1: Vascular adhesion molecule-1
XO: Xanthine oxidase.

## References

[1] Hoeper M. M., Bogaard H. J., Condliffe R. (2013). Definitions and diagnosis of pulmonary hypertension. *Journal of the American College of Cardiology*.

[2] Tabima D. M., Frizzell S., Gladwin M. T. (2012). Reactive oxygen and nitrogen species in pulmonary hypertension. *Free Radical Biology and Medicine*.

[3] Giaid A., Yanagisawa M., Langleben D. (1993). Expression of endothelin-1 in the lungs of patients with pulmonary hypertension. *New England Journal of Medicine*.

[4] Stewart D. J., Levy R. D., Cernacek P., Langleben D. (1991). Increased plasma endothelin-1 in pulmonary hypertension: marker or mediator of disease?. *Annals of Internal Medicine*.

[5] Adnot S., Raffestin B., Eddahibi S., Braquet P., Chabrier P.-E. (1991). Loss of endothelium-dependent relaxant activity in the pulmonary circulation of rats exposed to chronic hypoxia. *Journal of Clinical Investigation*.

[6] Humbert M., Morrell N. W., Archer S. L. (2004). Cellular and molecular pathobiology of pulmonary arterial hypertension. *Journal of the American College of Cardiology*.

[7] Kähler J., Ewert A., Weckmüller J. (2001). Oxidative stress increases endothelin-1 synthesis in human coronary artery smooth muscle cells. *Journal of Cardiovascular Pharmacology*.

[8] Kähler J., Mendel S., Weckmüller J. (2000). Oxidative stress increases synthesis of big endothelin-1 by activation of the endothelin-1 promoter. *Journal of Molecular and Cellular Cardiology*.

[9] Rafikova O., Rafikov R., Kumar S. (2013). Bosentan inhibits oxidative and nitrosative stress and rescues occlusive pulmonaryhypertension. *Free Radical Biology and Medicine*.

[10] Csiszar A., Labinskyy N., Olson S. (2009). Resveratrol prevents monocrotaline-induced pulmonary hypertension in rats. *Hypertension*.

[11] Jankov R. P., Kantores C., Pan J., Belik J. (2008). Contribution of xanthine oxidase-derived superoxide to chronic hypoxic pulmonary hypertension in neonatal rats. *American Journal of Physiology - Lung Cellular and Molecular Physiology*.

[12] Dubois M., Delannoy E., Duluc L. (2013). Biopterin metabolism and eNOS expression during hypoxic pulmonary hypertension in mice. *PLoS ONE*.

[13] Khoo J. P., Zhao L., Alp N. J. (2005). Pivotal role for endothelial tetrahydrobiopterin in pulmonary hypertension. *Circulation*.

[14] Brennan L. A., Steinhorn R. H., Wedgwood S. (2003). Increased superoxide generation is associated with pulmonary hypertension in fetal lambs: a role for NADPH oxidase. *Circulation Research*.

[15] Spiekermann S., Schenk K., Hoeper M. M. (2009). Increased xanthine oxidase activity in idiopathic pulmonary arterial hypertension. *European Respiratory Journal*.

[16] Bowers R., Cool C., Murphy R. C. (2004). Oxidative stress in severe pulmonary hypertension. *American Journal of Respiratory and Critical Care Medicine*.

[17] Münzel T., Daiber A., Gori T. (2011). Nitrate therapy: new aspects concerning molecular action and tolerance. *Circulation*.

[18] Packer M., Halperin J. L., Brooks K. M., Rothlauf E. B., Lee W. H. (1984). Nitroglycerin therapy in the management of pulmonary hypertensive disorders. *The American Journal of Medicine*.

[19] Krieg P., Wahlers T., Giess W. (1998). Inhaled nitric oxide and inhaled prostaglandin E1: effect on left ventricular contractility when used for treatment of experimental pulmonary hypertension. *European Journal of Cardio-thoracic Surgery*.

[20] Yurtseven N., Karaca P., Kaplan M. (2003). Effect of nitroglycetin inhalation on patients with pulmonary hypertension undergoing mitral valve replacement surgery. *Anesthesiology*.

[21] Pepke-Zaba J., Higenbottam T. W., Dinh-Xuan A. T., Stone D., Wallwork J. (1991). Inhaled nitric oxide as a cause of selective pulmonary vasodilatation in pulmonary hypertension. *The Lancet*.

[22] Sahara M., Sata M., Morita T., Hirata Y., Nagai R. (2012). Nicorandil attenuates monocrotaline-induced vascular endothelial damage and pulmonary arterial hypertension. *PLoS ONE*.

[23] Oelze M., Knorr M., Kröller-Schön S. (2013). Chronic therapy with isosorbide-5-mononitrate causes endothelial dysfunction, oxidative stress, and a marked increase in vascular endothelin-1 expression. *European Heart Journal*.

[24] Gori T., Daiber A. (2009). Non-hemodynamic effects of organic nitrates and the distinctive characteristics of pentaerithrityl tetranitrate. *American Journal of Cardiovascular Drugs*.

[25] Quinn M. J., Crouse L. C. B., McFarland C. A., Lafiandra E. M., Johnson M. S. (2009). Reproductive and developmental effects and physical and chemical properties of pentaerythritol tetranitrate (PETN) in the rat. *Birth Defects Research Part B—Developmental and Reproductive Toxicology*.

[26] Bucher J. R., Huff J., Haseman J. K., Eustis S. L., Lilja H. S., Murthy A. S. K. (1990). No evidence of toxicity or carcinogenicity of pentaerythritol tetranitrate given in the diet to F344 rats and B6C3F1 mice for up to two years. *Journal of Applied Toxicology*.

[27] Schuhmacher S., Oelze M., Bollmann F. (2011). Vascular dysfunction in experimental diabetes is improved by pentaerithrityl tetranitrate but not isosorbide-5-mononitrate therapy. *Diabetes*.

[28] Schuhmacher S., Wenzel P., Schulz E. (2010). Pentaerythritol tetranitrate improves angiotensin II-induced vascular dysfunction via induction of heme oxygenase-1. *Hypertension*.

[29] Oppermann M., Balz V., Adams V. (2009). Pharmacological induction of vascular extracellular superoxide dismutase expression in vivo. *Journal of Cellular and Molecular Medicine*.

[30] Daiber A., Oelze M., Wenzel P., Bollmann F., Pautz A., Kleinert H. (2012). Heme oxygenase-1 induction and organic nitrate therapy: beneficial effects on endothelial dysfunction, nitrate tolerance, and vascular oxidative stress. *International Journal of Hypertension*.

[31] Wenzel P., Oelze M., Coldewey M. (2007). Heme oxygenase-1: a novel key player in the development of tolerance in response to organic nitrates. *Arteriosclerosis, Thrombosis, and Vascular Biology*.

[32] Oberle S., Schwartz P., Abate A., Schröder H. (1999). The antioxidant defense protein ferritin is a novel and specific target for pentaerithrityl tetranitrate in endothelial cells. *Biochemical and Biophysical Research Communications*.

[33] Shimzu K., Takahashi T., Iwasaki T. (2008). Hemin treatment abrogates monocrotaline-induced pulmonary hypertension. *Medicinal Chemistry*.

[34] Christou H., Morita T., Hsieh C.-M. (2000). Prevention of hypoxia-induced pulmonary hypertension by enhancement of endogenous heme oxygenase-1 in the rat. *Circulation Research*.

[35] Münzel T., Giaid A., Kurz S., Stewart D. J., Harrison D. G. (1995). Evidence for a role of endothelin 1 and protein kinase C in nitroglycerin tolerance. *Proceedings of the National Academy of Sciences of the United States of America*.

[36] Oelze M., Kröller-Schön S., Steven S. (2014). Glutathione peroxidase-1 deficiency potentiates dysregulatory modifications of endothelial nitric oxide synthase and vascular dysfunction in aging. *Hypertension*.

[37] Meloan S. N., Puchtler H. (1987). “Harris hematoxylin,” what harris really wrote and the mechanism of hemalum stains. *Journal of Histotechnology*.

[38] Urboniene D., Haber I., Fang Y.-H., Thenappan T., Archer S. L. (2010). Validation of high-resolution echocardiography and magnetic resonance imaging vs. high-fidelity catheterization in experimental pulmonary hypertension. *American Journal of Physiology—Lung Cellular and Molecular Physiology*.

[39] Daiber A., August M., Baldus S. (2004). Measurement of NAD(P)H oxidase-derived superoxide with the luminol analogue L-012. *Free Radical Biology and Medicine*.

[40] Wenzel P., Schulz E., Oelze M. (2008). AT1-receptor blockade by telmisartan upregulates GTP-cyclohydrolase I and protects eNOS in diabetic rats. *Free Radical Biology and Medicine*.

[41] Wendt M. C., Daiber A., Kleschyov A. L. (2005). Differential effects of diabetes on the expression of the gp91^phox^ homologues nox1 and nox4. *Free Radical Biology and Medicine*.

[42] Hausding M., Jurk K., Daub S. (2013). CD40L contributes to angiotensin II-induced pro-thrombotic state, vascular inflammation, oxidative stress and endothelial dysfunction. *Basic Research in Cardiology*.

[43] Rosenkranz S. (2015). Pulmonary hypertension 2015: current definitions, terminology, and novel treatment options. *Clinical Research in Cardiology*.

[44] Predel H.-G., Knigge H., Prinz U., Kramer H. J., Stalleicken D., Rost R. E. (1995). Exercise increases endothelin-1 plasma concentrations in patients with coronary artery disease: modulatory role of LDL cholesterol and of pentaerithrityltetranitrate. *Journal of Cardiovascular Pharmacology*.

[45] Gomez-Arroyo J. G., Farkas L., Alhussaini A. A. (2012). The monocrotaline model of pulmonary hypertension in perspective. *American Journal of Physiology—Lung Cellular and Molecular Physiology*.

[46] Chen M.-J., Chiang L. Y., Lai Y.-L. (2001). Reactive oxygen species and substance P in monocrotaline-induced pulmonary hypertension. *Toxicology and Applied Pharmacology*.

[47] DeMarco V. G., Whaley-Connell A. T., Sowers J. R., Habibi J., Dellsperger K. C. (2010). Contribution of oxidative stress to pulmonary arterial hypertension. *World Journal of Cardiology*.

[48] Xu D., Guo H., Xu X. (2011). Exacerbated pulmonary arterial hypertension and right ventricular hypertrophy in animals with loss of function of extracellular superoxide dismutase. *Hypertension*.

[49] Daiber A., Münzel T. (2015). Organic nitrate therapy, nitrate tolerance, and nitrate-induced endothelial dysfunction: emphasis on redox biology and oxidative stress. *Antioxidants and Redox Signaling*.

[50] Münzel T., Daiber A., Gori T. (2013). More answers to the still unresolved question of nitrate tolerance. *European Heart Journal*.

[51] Jurt U., Gori T., Ravandi A., Babaei S., Zeman P., Parker J. D. (2001). Differential effects of pentaerythritol tetranitrate and nitroglycerin on the development of tolerance and evidence of lipid peroxidation: A Human In Vivo Study. *Journal of the American College of Cardiology*.

[52] Gori T., Al-Hesayen A., Jolliffe C., Parker J. D. (2003). Comparison of the effects of pentaerythritol tetranitrate and nitroglycerin on endothelium-dependent vasorelaxation in male volunteers. *American Journal of Cardiology*.

[53] Schnorbus B., Schiewe R., Ostad M. A. (2010). Effects of pentaerythritol tetranitrate on endothelial function in coronary artery disease: Results of the PENTA Study. *Clinical Research in Cardiology*.

[54] Hacker A., Müller S., Meyer W., Kojda G. (2001). The nitric oxide donor pentaerythritol tetranitrate can preserve endothelial function in established atherosclerosis. *British Journal of Pharmacology*.

[55] Oberle S., Abate A., Grosser N. (2003). Endothelial protection by pentaerithrityl trinitrate: bilirubin and carbon monoxide as possible mediators. *Experimental Biology and Medicine*.

[56] Wenzel P., Rossmann H., Müller C. (2015). Heme oxygenase-1 suppresses a pro-inflammatory phenotype in monocytes and determines endothelial function and arterial hypertension in mice and humans. *European Heart Journal*.

[57] Shen Y., Ward N. C., Hodgson J. M. (2013). Dietary quercetin attenuates oxidant-induced endothelial dysfunction and atherosclerosis in apolipoprotein e knockout mice fed a high-fat diet: a critical role for heme oxygenase-1. *Free Radical Biology and Medicine*.

[58] Yu J. C., Jae Y. H., Sang G. L. (2009). Temporal changes of angiopoietins and Tie2 expression in rat lungs after monocrotaline-induced pulmonary hypertension. *Comparative Medicine*.

